# Plasma Effects on Properties and Structure of Corn Starch: Characterization and Analysis

**DOI:** 10.3390/foods12214042

**Published:** 2023-11-06

**Authors:** Cunshe Chen, Fang Tong, Ruohao Sun, Jufang Yang, Zhihua Pang, Xinqi Liu

**Affiliations:** China Food Flavor and Nutrition Health Innovation Center, Beijing Technology and Business University, Beijing 100083, China; chencs@th.btbu.edu.cn (C.C.); tongfang1522@163.com (F.T.); sunrh0310@163.com (R.S.); yjf13792@163.com (J.Y.); liuxinqi@btbu.edu.cn (X.L.)

**Keywords:** corn starch, plasma, performance characterization

## Abstract

This research investigated the impact of air plasma and high-pressure plasma treatments on corn starch. The resulting samples were characterized by particle morphology, molecular polymerization degree, molecular functional groups, and crystallinity. SEM analysis revealed that plasma treatment altered the surface morphology of corn starch, with variations observed depending on the duration of treatment. UV/Vis spectroscopy results indicated that longer plasma exposure times increased maximum absorbance values with less complete peak shapes. FTIR results demonstrated that plasma treatment disrupted the crystalline structure of starch, resulting in decreased molecular polymerization. Lastly, XRD results showed a proportional relationship between plasma treatment duration and the intensity of the diffuse peak, indicating that prolonged plasma exposure increased the amorphous nature of starch.

## 1. Introduction

Starch serves as the primary source of carbohydrates in the human diet, offering essential energy and playing roles as a bulking agent, thickener, gelling agent, and water retention enhancer [1]. It holds significant industrial value and finds widespread application in the food, medicine, and cosmetic industries [2]. The special structure of starch determines its particular properties. Generally, the ratio of amylose to amylopectin in natural starch is about 1:3, and in addition, it also contains a small amount of minerals and phosphate esters. However, due to the intricate spatial arrangement involving straight-chain and branched-chain starch, starch exhibits a highly complex multi-scale structure. Generally, the ratio of amylose to amylopectin in natural starch is about 1:3, and in addition, it also contains a small amount of minerals and phosphate esters. Native starch is characterized by low solubility, inadequate shear and thermal stability, and poor physical and functional properties, which limits its application and development in various industrial sectors [3]. Researchers and scholars have pointed out that starch modification presents a significant market opportunity to meet the demand for novel functional and value-added properties of modified starch [4]. Consequently, current research in starch predominantly focuses on modified starch, employing various physical, chemical, enzymatic, and compound modifications [5]. After different treatments, microcrystalline starch with certain structures and properties can be obtained, and its properties are also changed.

Plasma technology is used in the field of food because of its simple operation, green, pollution-free nature, and ability to save time and effort. Current research on plasma starch mainly includes the following aspects: one is the use of plasma technology to improve starch’s solubility; the second is the use of plasma technology and starch grafting, the cross-linking or depolymerization reaction, which changes the structure and characteristics of starch; and third is the use of plasma-processing starch to destroy its chemical bond and crystal structure and change the structure and characteristics of natural starch so as to achieve the purpose of obtaining modified starch. Plasma, an ionized gas containing a wide variety of active particles, such as electrons, positive and negative ions, free radicals, atoms, and excited or non-excited molecules, possesses the energy required to disturb covalent bonds and initiate various reactions, resulting in the formation of volatile compounds [6,7,8]. In the process of plasma collision, there is an energy change when the number of positive and negative ions is equal and the whole is electroneutral. Plasma is currently an emerging green technology in the food sector, offering several advantages, including preserving the nutritional, functional, and sensory properties of food products, ensuring microbiological safety, and maintaining the fresh appearance of food products [9,10]. With its high efficiency, low energy consumption, and minimal side reactions [11], plasma technology has a good application prospect in starch modification. The changes in plasma-treated starch in its microstructure, including crosslinking and depolymerization within molecules and functional groups, improve the properties of natural starch. Currently, research on plasma-modified starch is also relatively popular. For instance, atmospheric pressure cold plasma has demonstrated the selective modification of protein structures, thereby improving whey protein separation [12]. Additionally, air-cold plasma treatment has been found to increase the water and fat-binding capabilities of protein-rich pea flour [13]. Low-pressure plasma treatments have been observed to improve the germination rate, seedling length, water absorption capacity, and the levels of α-amylase activity and γ-aminobutyric acid in germinated brown rice [14].

This study investigates the structural characteristics of corn starch using two distinct plasma treatments as follows: air plasma and low-temperature, low-pressure plasma, under various treatment durations. On the one hand, it guarantees microbial safety in the process of starch modification, and a green way is adopted to enhance the availability of starch and broaden the application scope of starch. On the other hand, it also expands the application scope of plasma technology and broadens its application prospects in the field of food. The molecular structure and functional properties of corn starch were elucidated under these two different plasma treatments, providing valuable insights into the potential application of plasma technology in starch modification.

## 2. Materials and Methods

### 2.1. Materials and Reagents

Crystalline iodine, potassium iodide, citric acid monohydrate, and disodium hydrogen phosphate dodecahydrate were all of the analytical grade and sourced from the Beijing Chemical Factory. Soluble starch of an analytical grade was obtained from Beijing Auboxin Biotechnology Co., Ltd. (Beijing, China).

### 2.2. Instruments and Equipment

Plasma generator type A (Sanfish Electric Equipment Co., Ltd., Hangzhou, China), UV-visible spectrophotometer UV2550 (Shimadzu Japan, Chengdu, China), scanning electron microscope Model TESCAN VEGA II (TESCAN, S.r.o. Co., Ltd., Shanghai, China), Fourier transform infrared (FTIR) spectrometer Model Avatar 370 (Nicolet, Madison, WI, USA), X-ray Diffraction Spectrometer Model XRD-6000 (Shimadzu Japan, Chengdu, China), High-Speed Refrigerated Centrifuge Model Himac CR 22G (Hitachi Limited, Tokyo, Japan), Digital Constant Temperature Water Bath Model HH-2 (Jiangsu Jierui Electric Co., Ltd., Nanjing, China), and Electronic Analytical Balance Model JA5003 (Shanghai Precision Scientific Instruments Co., Shanghai, China). A test tube, triangle bottle, beaker, volumetric flask, centrifuge tube, glass bar, timer, cuvette, and four layers of gauze were all the instruments used in this experiment.

### 2.3. Methods

#### 2.3.1. Plasma Treatment of Starch Samples

1. Air plasma action starch solid powder sample: weigh 0.100 g of starch in the ampere tube; use spark discharge excitation to produce air plasma action on starch; set the action time to 0 s, 30 s, 60 s, 90 s, 120 s, 150 s, 180 s, and 300 s; and perform three parallel experiments at each time point. Also, to reduce the thermal effect of plasma produced by excessive heat on starch using the 10 s interval discharge method, starch samples with different plasma action times were obtained. Put the samples after action into the dryer for analysis and testing.
2. Low-temperature and low-pressure plasma starch solid powder sample: weigh 0.100 g of starch in amp tubes and plug skimmed cotton in the seal to prevent the starch powder from sucking during the vacuum; connect the ampere with starch to the vacuum system; spark discharge to generate plasma at 0 s, 30 s, 60 s, 90 s, 120 s, 150 s, 180 s, and 300 s; and perform three parallel experiments at each time point. To reduce the thermal effect of plasma, the effect of excessive heat on the 10 s interval was measured. The starch samples with different plasma action times were obtained. Put the samples after action into the dryer for analysis and testing.

#### 2.3.2. Characterization of Starch Properties

Starch is a polysaccharide polymer compound formed by the polymerization of glucose with the chemical formula C_6_H_12_O_6_. It consists of α-D-(+) dehydrated glucose basic units. Starch can be divided into two categories as follows: straight-chain starch and branched-chain starch. The former has a branchless helical structure, while the latter consists of 24–30 glucose residues linked head-to-tail by α-1,4-glycosidic bonds with α-1,6-glycosidic bonds at the branched chains. Spectroscopy was used to characterize the functional groups and structures of the glucose units of starch and to explore the structural changes in starch before and after plasma treatment.

#### 2.3.3. Analysis of Sample Particle Morphology

As the starch under examination existed in a powdered form, the prepared samples were placed inside the scanning electron microscope’s sample chamber. Creating a vacuum environment is essential to prevent the powder particles from dispersing, contaminating the scanning electron microscope’s chamber, or compromising the quality of analysis. Therefore, the adhesive paper tape method was employed for sample preparation. The prepared sample was then inserted into the sample compartment of the ion-sputtering instrument, where they were subjected to a 90 s gold coating process under a current of 15 mA. Subsequently, the sample was removed and loaded into the observation chamber of the scanning electron microscope for analysis. The starch samples intended for electron microscopy observation were categorized into two groups as follows: solid powder samples subjected directly to air plasma treatment and samples that underwent low-pressure, low-temperature plasma treatment [15].

#### 2.3.4. Analysis of Molecular Polymerization Degree

The complex formed by starch and iodine was subjected to full-wavelength UV scanning to compare the changes in its maximum absorption wavelength and absorption intensity before and after the plasma treatment and to examine the effect of plasma on its molecular polymerization. Scanning was conducted over a wavelength range of 200 to 900 nm [16].

#### 2.3.5. Analysis of Molecular Groups

A small quantity of the dried sample was mixed with KBr powder. Under the illumination of an infrared lamp, this mixture was ground in an agate mortar for 2 to 5 min to ensure thorough homogenization. Subsequently, a small portion of the ground mixture powder was pressed into pellets and placed in the sample slot inside a Fourier transform infrared spectrometer. Full-wavelength scanning was performed within the 500 to 4500 cm^−1^ range, generating infrared spectra [17].

#### 2.3.6. Analysis of Molecular Crystalline Structure

The plasma treatment was applied to the starch sample for various durations (0, 1, 2, 3, 4, and 5 min), and the treated sample powder was used to create an X-ray diffraction sample. The analysis conditions were as follows: Cu Kα characteristic X-rays, copper target, graphite monochromator, voltage set at 40 KV, the emission and anti-reflection slit set at 1°, the receiving slit at 0.3 mm, scanning speed at 1.5 °/min, scanning range at 2θ = 10–65°, and scanning speed at 2 °/min [18].

## 3. Results and Discussion

### 3.1. Scanning Electron Microscope Analysis of Starch

**Figure 1:**
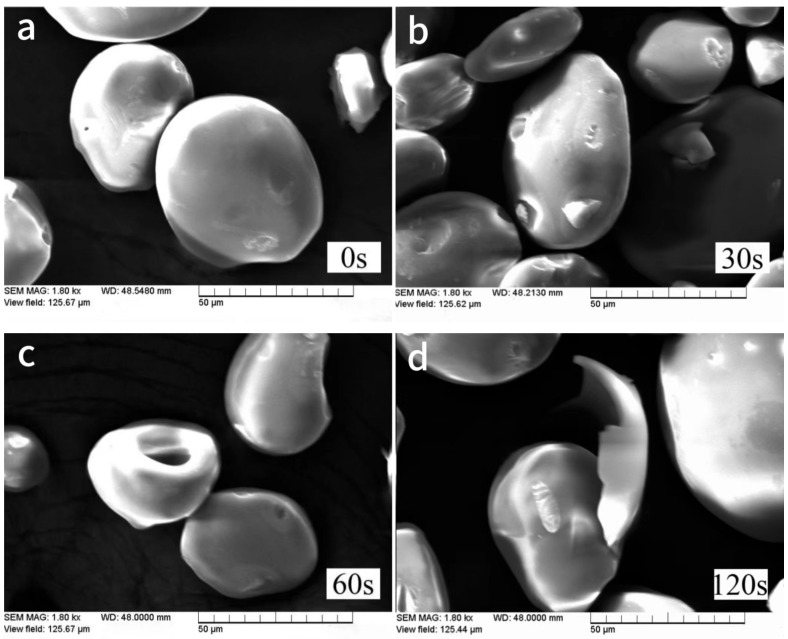
Scanning Electron Microscopy Images of starch treated with air plasma. The four images (**a**–**d**) represent SEM images without air plasma, air plasma for 30 s, 60 s for air plasma, and 120 s for air plasma, respectively.

**Figure 2:**
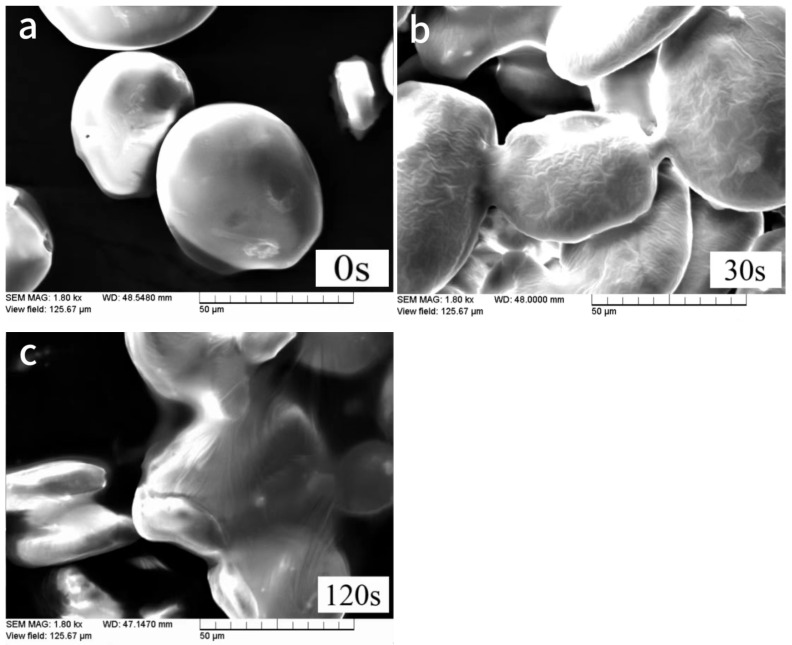
Scanning Electron Microscopy Images of starch treated with low-temperature and low-pressure plasma. The three images (**a**–**c**) represent SEM images without low voltage plasma, low pressure plasma for 30 s, and low pressure plasma for 120 s.

The size and shape of starch particles can also be used to distinguish the starch species. The morphology and structure of starch are affected by a variety of physical and chemical factors, and the size and shape of natural starch particles after plasma treatment is changed, so the mechanism of plasma action on starch can be inferred by analyzing the change in the structure of starch. Corn starch samples treated with air plasma and low-temperature, low-pressure plasma were subjected to scanning electron microscopy (SEM) to observe and capture representative images of the starch particle morphology. The objective was to investigate the impact of plasma treatment on the starch particle morphology. The results are illustrated in Figure 1 and Figure 2.

Figure 1 shows that the surface morphology of starch undergoes changes when subjected to the action of air plasma, and these changes become more pronounced with increasing treatment time. The four images represent the control, 30 s, 60 s, and 120 s plasma treatment on starch. Untreated starch granules are nearly spherical, with smooth and dense surfaces with no small pores or cracks, and are mostly dispersed. In addition, undamaged depressions appear on the surface of some starch granules. A possible reason for this is that the growth of starch granules is hindered by spherical protein microbodies, leaving growth traces [19]. After 30 s of exposure to air plasma, starch particles begin to show damage and microporosity, and some particles are eroded, exposing the layered structure inside the starch. When the treatment time with plasma reaches 60 s, the surfaces of starch particles become severely depressed, and holes appear. Thirumdas, Trimukhe, et al. [20] also found cracks and cavities on the surface of rice starch particles treated with air plasma. When the treatment time with air plasma was 2 min, it could be observed that part of the starch’s spherical structure had been completely destroyed, resulting in flaky and irregular particles, and the number of plasma-damaged particles increased sharply. A possible reason for this is that after the appearance of holes in starch particles, active plasma substances penetrate the interior of the starch particles through the cracks, causing starch depolymerization and fracture [15]. Plasma etching leads to the depolymerization of starch molecules, increasing the effective surface area of starch particles and increasing solubility compared to natural starch [17]. This modification of starch through plasma treatment makes it more suitable for practical production and processing. These surface changes also increase the sensitivity to enzymes and other chemical reactions.

Based on Figure 2, it is evident that low-pressure, low-temperature plasma can also influence the morphology of starch particles. After exposure to the two plasma conditions, the size of starch particles remained nearly consistent, but their morphology had notable differences. In Figure 2, from left to right, we have the control group, plasma exposure for 30 s, and plasma exposure for 120 s, as observed under a scanning electron microscope. After 30 s of exposure to low-pressure, low-temperature plasma, significant changes occurred in the starch particles. Firstly, a film-like layer formed on the surface of the particles, causing previously smooth surfaces to become wrinkled. The particles gradually transited from spherical to flat while maintaining their structural integrity. Secondly, the originally dispersed particles aggregated and cross-linked, forming membrane channels between particles. At this point, starch particles aggregated and created a unified outer shell layer among multiple molecules. When exposed to low-pressure, low-temperature plasma for 120 s, the aggregation and cross-linking between particles became even more pronounced. This process somewhat makes starch more susceptible to degradation via α-amylase. Studies have shown that low-pressure, low-temperature plasma can improve enzyme activity and become more effective than the activation of air plasma. This figure illustrates that the surface of starch particles is affected by the duration of plasma exposure, with longer exposure times leading to more pronounced changes in particle morphology. This finding is consistent with the research conducted by Thirumdas, Deshmukh et al. [19]. Furthermore, some studies have found that high-intensity plasma can also increase the surface damage of starch particles [21,22].

### 3.2. Ultraviolet Spectral Analysis of the Complex Formed by Starch and Iodine

**Figure 3:**
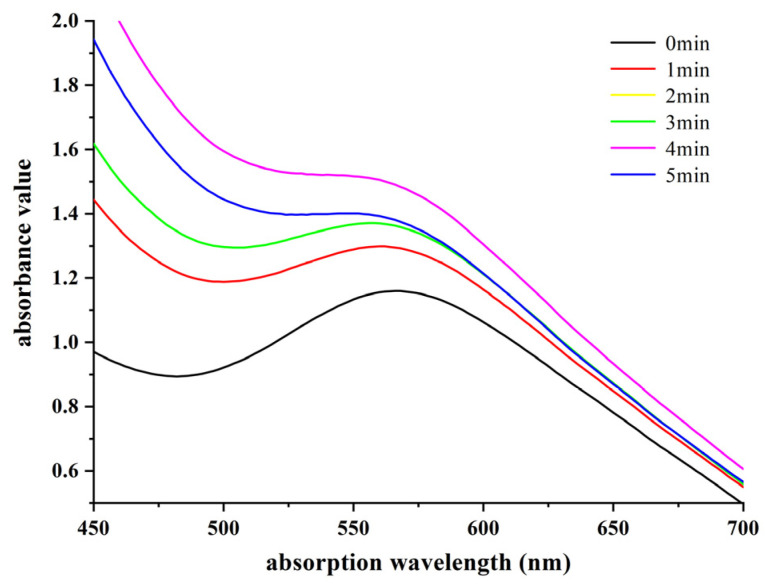
The effect of plasma on the UV absorption of the starch-iodine complex.

Starch and iodine complexate and form colored complexes, and different colors correspond to different maximum absorption wavelengths. The UV-visible absorption spectrum is the absorption spectrum generated by the ultraviolet–visible light region (200~800 nm) causing the energy level transition, which can reflect the maximum absorption wavelength and wavelength range of the complex. After the action of plasma, starch produces an unsaturated structure, and the absorption of ultraviolet light makes the starch and iodine complex react. Therefore, the UV-visible absorption spectrum reflects the structural characteristics of starch and, to some extent, some of the properties of starch. According to the method outlined in Section 2.3.4, starch samples subjected to plasma treatment were subjected to full-wavelength UV scanning. The aim was to compare the changes in the maximum absorption wavelength and the maximum absorbance photometric value of the complex formed between starch and iodine before and after plasma treatment. This qualitative assessment was conducted to investigate the impact of plasma treatment on the starch composition, and the results are presented in Figure 3.

As can be seen from Figure 3, the maximum absorption wavelength of the complex formed by starch and iodine was 567 nm, and the maximum absorption wavelength of the complex formed with starch shifted to the left while the maximum absorption wavelength decreased. A possible reason for this is that when iodine comes into contact with starch—iodine molecules into the helix of starch molecules—the formation of a dark, greater UV absorption, and amylopectin on each branch’s average length is shorter, the corresponding number of complexed iodine molecules is less, and under the same conditions UV absorption is smaller [16,23,24]. Secondly, the maximum absorbance luminosity value of all plasma sample groups is strengthened, and the longer the action time, the larger the proportion of maximum absorbance values increase, while the peak type is less complete. A possible reason for this is that plasma breaks the long chain structure of starch molecules, the content of amylopectin is reduced into more amylose, and the ability of complexed iodine molecules is gradually increased [25]. Therefore, plasma changes the content of straight chain and amylopectin in starch molecules to the point that the hydrolysis reaction is more rapid and thorough under the same time and conditions.

### 3.3. Infrared Spectral Analysis of Starch

**Figure 4:**
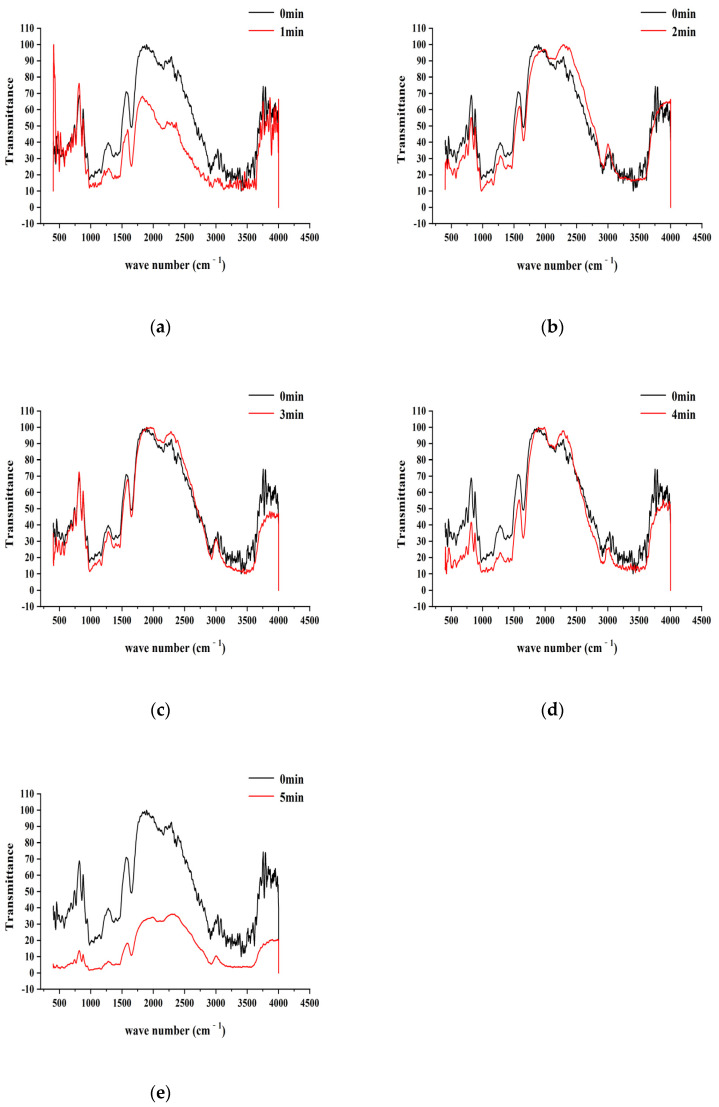
The infrared spectra figures of starch affected by air plasma. (**a**–**e**) represent the corresponding FTIR images of the air plasma treatment of 1, 2, 3, 4, and 5 min, respectively.

**Figure 5:**
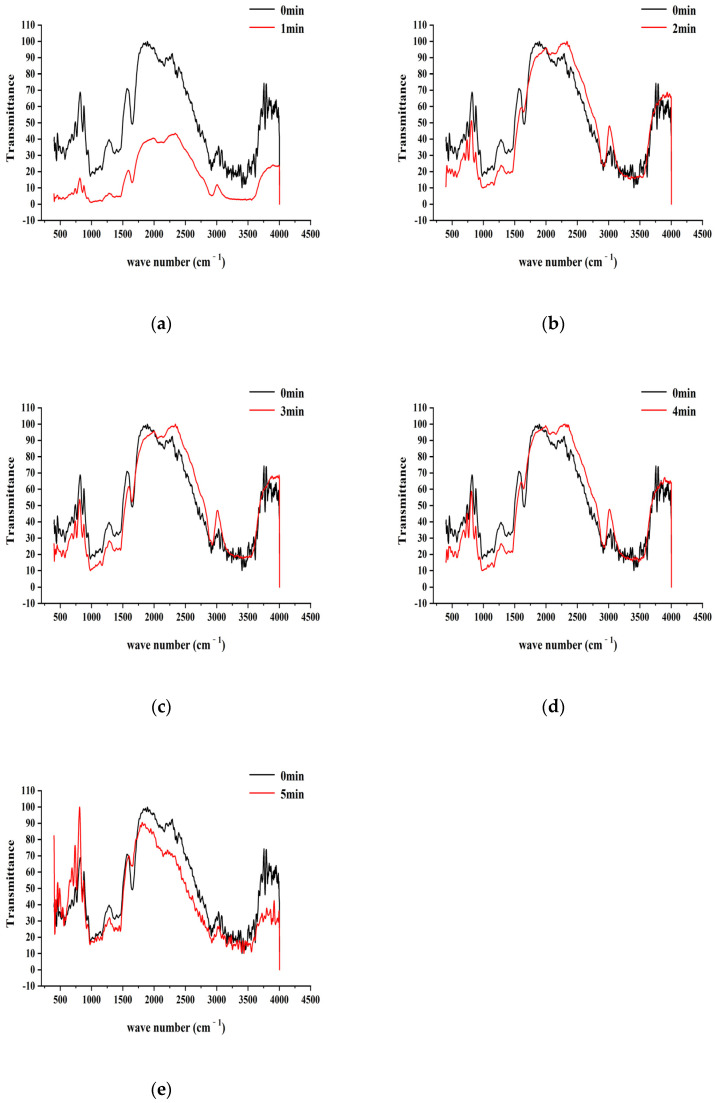
The infrared spectra of starch affected by low-pressure, low-temperature plasma. (**a**–**e**) represent the corresponding FTIR images treated at 1, 2, 3, 4, and 5 min, respectively.

As a method of infrared spectroscopy to characterize and identify compounds, it produces energy level transitions according to the selective absorption degree of radiation in the infrared light region, according to the waveform, intensity, position, and the number of peaks of the spectrum, and is often used to analyze the structure and chemical bond properties of molecules. Infrared spectroscopy is highly specific. In this experiment, a Fourier Infrared Spectrometer was used to analyze the change in the infrared absorption of starch before and after the action of air and low-temperature plasma and to explore the influence of plasma on the molecular group and crystallinity of the sample. There are many methods for infrared spectrometers, and the method in this experiment was the KBr pressing method. Following the procedure outlined in Section 2.3.5, Fourier-transform infrared (FTIR) spectroscopy was employed to analyze the structure and functional groups of starch molecules that had undergone plasma treatment. This analysis aimed to characterize the changes in characteristic structures and functional groups on the molecular units of starch. The results are shown in Figure 4 and Figure 5.

Figure 4 and Figure 5 represent the Fourier-transform infrared spectra of starch after treatment with air plasma and low-pressure, low-temperature plasma, respectively. Each system includes one control group (original starch) and five sample groups corresponding to plasma treatment times of 0, 1, 2, 3, 4, and 5 min. From the infrared spectra of the control group, it is evident that starch exhibits characteristic absorption peaks at 3404 cm^−1^, 2914 cm^−1^, 1655 cm^−1^, and 1163 cm^−1^, representing stretching vibrations of O-H bonds involved in hydrogen bonding, asymmetric stretching vibrations of C-CH2-C, bending vibrations of water molecules, and asymmetric stretching motions of C-O-C, respectively [17].

Figure 4 reveals that the positions of the absorption peaks of characteristic functional groups in starch treated with air plasma show no significant differences, and there are no new absorption peaks in the spectra, indicating that plasma alters the secondary and higher-order structures of starch within a limited range through physicochemical processes [26]. However, the intensities of absorption peaks at the same wavenumber change; specifically, all characteristic absorption peaks decrease after 1 min of treatment. However, peak intensities show a slight recovery trend from 2 to 4 min of plasma treatment. The absorption intensity decreases sharply when the plasma treatment time reaches 5 min, suggesting the significant disruption of starch’s crystalline structure, a decrease in molecular polymerization, and an increase in amorphousness [18].

Figure 5 reveals that, unlike air plasma, low-pressure, low-temperature plasma affected the intensity of characteristic peaks and resulted in the disappearance of peaks at specific wavenumbers. Compared to the control group, the absorption peak at 3404 cm^−1^ weakened or disappeared, indicating that plasma weakened the stretching vibration of O-H bonds involved in hydrogen bonding. It could also be due to a feedback inhibition effect, where hydroxyl groups generated by the plasma itself interact with the hydroxyl groups in starch molecules [27]. The intensities of the absorption peaks at 1655 cm^−1^ and 1163 cm^−1^ were lower than those in the control group, and the area of the C-O-C peak decreased, indicating starch depolymerization. At this point, the plasma disrupts the crystalline structure of starch, leading to increased starch reactivity [28].

### 3.4. X-ray Diffraction Spectral Analysis of Starch Molecules Crystallinity

**Figure 6:**
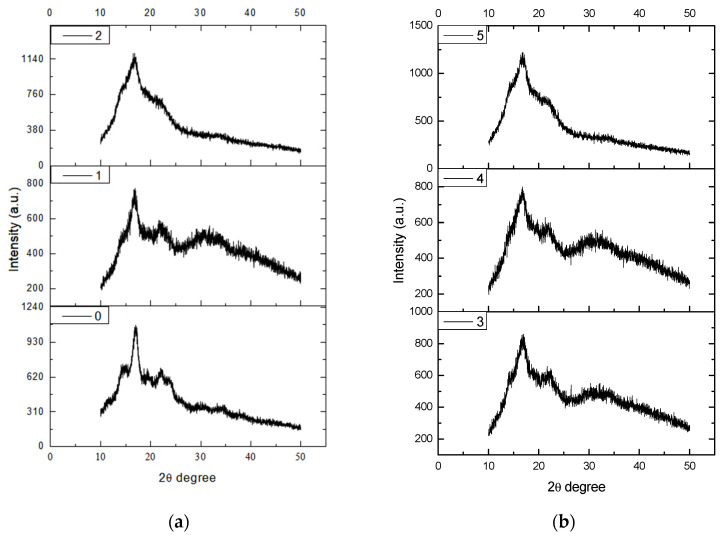
X-ray diffraction spectra of starch treated by air plasma. The corresponding X-ray images of the air-plasma-treated starch at 0, 1, 2, 3, 4, and 5 min are shown in Figure. (**a**) shows the X-ray diffraction pattern processed for 0, 1, and 2 min with air-plasma-treated, and (**b**) shows the X-ray diffraction pattern processed with air-plasma-treated for 3, 4, and 5 min.

**Figure 7:**
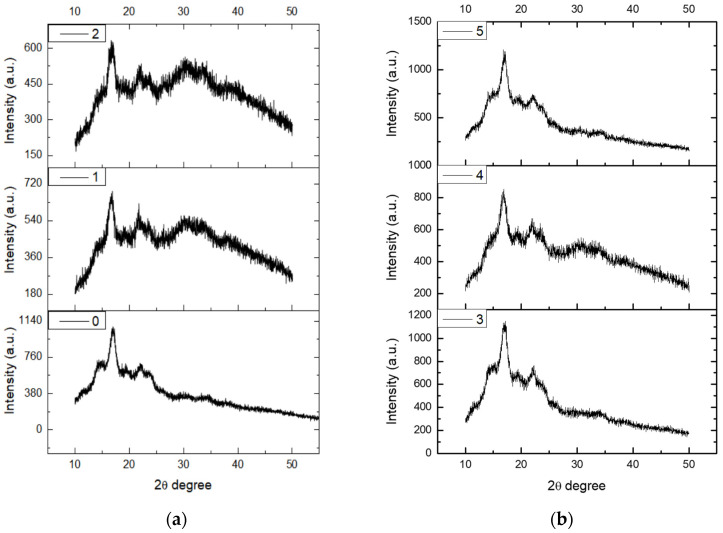
X-ray diffraction spectra of starch treated by low-pressure, low-temperature plasma. The corresponding X-ray images of the low-pressure, low-temperature plasma treated starch at 0, 1, 2, 3, 4, and 5 min are shown in Figure. (**a**) shows the X-ray diffraction pattern processed for 0, 1, and 2 min with low-pressure, low-temperature plasma, and (**b**) shows the X-ray diffraction pattern processed with low-pressure, low-temperature plasma for 3, 4, and 5 min.

X-ray diffraction technology is mainly used to analyze the long-range ordered structure of starch particles. In the process of starch granule biosynthesis, circadian photosynthesis is very different, so the rate of glucose transfer to cells is also very different. The crystallization area and amorphous area appear alternately, making the internal density of starch different to produce a “growth ring”. X-ray diffraction, when applied to plasma starch, can analyze the crystallinity and type of crystallization of starch molecules. Following the procedure outlined in Section 2.3.6, X-ray diffraction spectroscopy was employed to analyze the crystallinity of starch molecules after their exposure to plasma. This analysis aimed to investigate the impact of plasma on the structural properties of starch. The experimental results are shown in Figure 6 and Figure 7.

Figure 6 and Figure 7 represent the X-ray diffraction patterns of starch after treatment with air plasma and low-pressure, low-temperature plasma, respectively. From the figures, it is evident that the structure of the original starch consisted of crystalline and amorphous regions. At a diffraction angle of 2θ = 16.989°, there was a maximum absorption peak in the diffraction curve, which is a characteristic peak of starch with an intensity of 1082 a.u.

As can be seen from Figure 6, the intensities of the absorption peaks at the diffraction angle 2θ = 16.989° for the five sample groups treated with air plasma for 1 to 5 min were 596, 560, 1135, 768, and 1079, respectively. Similarly, besides enhancing diffraction signals at 3 and 5 min of plasma treatment, the diffraction signals at other time points showed a sharp decrease relative to the original starch. This suggests that air plasma has not completely disrupted the crystalline structure of starch, and the diffraction angle for the characteristic diffraction peak of starch remains unchanged. Bie et al. [29] reported similar findings in their study of cold plasma-treated cassava starch. The amorphous region was more severely damaged during plasma treatment than the crystalline region. This could be because plasma can only penetrate a few nanometers into the starch particles. While the signals of sharp diffraction peaks increased at 3 min and 5 min, the signals of the diffuse peaks corresponding to the other sample groups were weaker. This indicates that the increase in crystallinity is also due to the change in orientation of straight-chain starch composed of double helices inside the crystal. A possible explanation for this is that plasma treatment did not produce new substances in starch but only transformed the internal structure of starch between crystalline and amorphous states.

Figure 7 shows that low-pressure, low-temperature plasma of a certain intensity can alter the crystalline characteristics of starch, and this change in crystallinity is related to the plasma treatment time. For the five sample groups with plasma treatment times of 1 to 5 min, the absorption peak intensities at the diffraction angle 2θ = 16.989° were 758, 1130, 777, 763, and 1065, respectively. Analysis showed that the plasma treatment time was directly proportional to the intensity of the diffuse peak. In other words, an extended plasma treatment time increases the amorphousness of starch. Additionally, it can be inferred that, overall, the plasma treatment weakens the intensity of the characteristic sharp diffraction features of starch while enhancing the intensity of diffuse diffraction features.

## 4. Conclusions and Recommendations

This study demonstrates that both air plasma and low-temperature, low-pressure plasma treatments have a certain impact on the structural properties of microcrystalline starch. The active substances in the plasma-induced depolymerization of starch chains, resulting in etching on the particle surface and a decrease in the degree of molecular polymerization, led to a reduction in relative crystallinity and an increase in the amorphousness of starch. Importantly, it should be noted that these plasma treatments do not induce any alterations in the crystalline polymorph of starch, nor do they facilitate the generation of novel substances. Instead, their principal effect lies in inducing the transformation of the internal structural configuration of starch molecules between crystalline and amorphous states.

This study provides a theoretical foundation for applying air plasma, low-temperature, and low-pressure plasma technologies in the modification of starch. However, research on some starch properties remains incomplete, necessitating further investigative strides in the future. Therefore, plasma technology, as an innovative approach to starch modification, is anticipated to attract increased attention.

## Data Availability

The data used to support the findings of this study can be made available by the corresponding author upon request.
